# Supplementary material for: Chiral coordination polymer nanowires boost radiation-induced in situ tumor vaccination
Source: Nat Commun. 2024 May 9;15:3902. doi: 10.1038/s41467-024-48423-w (PMC11082158; doi:10.1038/s41467-024-48423-w)
Supplement: Supplementary file 1 — Supplementary Information [file 41467_2024_48423_MOESM1_ESM.pdf]

Supplementary Information

Chiral coordination polymer nanowires boost radiation-induced in situ tumor vaccination

Zhusheng Huang[1,2,3], Rong Gu[1], Shiqian Huang[2], Qian Chen[1], Jing Yan[4], Xiaoya Cui[5], Haojie Jiang[1], Dan Yao[1], Chuang Shen[2], Jiayue Su[5], Tao Liu[5], Jinhui Wu[1], Zhimin Luo[2,*], Yiqiao Hu[1,*] and Ahu Yuan[1,*]

[1] State Key Laboratory of Pharmaceutical Biotechnology, Medical School and School of Life Science, Nanjing University, Nanjing 210093, China.

[2] State Key Laboratory for Organic Electronics and Information Displays (SKLOEID), School of Chemistry and Life Sciences, Nanjing University of Posts and Telecommunications, Nanjing 210023, China.

[3] Cancer Centre and Institute of Translational Medicine, Faculty of Health Sciences, University of Macau, Macau SAR 999078, China.

[4] The Comprehensive Cancer Centre of Drum Tower Hospital, Medical School of Nanjing University, Nanjing 210023, China.

[5] Beijing Frontier Research Center for Biological Structures, School of Life Sciences, Tsinghua University, Beijing 100083, China.

These authors contributed equally: Zhusheng Huang, Rong Gu, Shiqian Huang

These authors jointly supervised this work: Zhimin Luo, Yiqiao Hu, Ahu Yuan

[*]Corresponding author's E-mail: iamzmluo@njupt.edu.cn (Z. L.), huyiqiao@nju.edu.cn (Y.H.) and yuannju@nju.edu.cn (A.Y.).

Inventory of Supporting Information

Supplementary Figure 1. Preparation and characterization of aAGd-NWs.

Supplementary Figure 2. Cryo-EM analysis of aAGd-NWs.

Supplementary Figure 3. Radiosensitization of aAGd-NWs in vitro.

Supplementary Figure 4. Flow cytometry analysis of apoptosis.

Supplementary Figure 5. Flow cytometry analysis of 3D tumor spheroids.

Supplementary Figure 6. The biodistribution of aAGd-NWs.

Supplementary Figure 7. Radiosensitization efficacy evaluation in vivo.

Supplementary Figure 8. Immunofluorescence and immunohistochemical staining.

Supplementary Figure 9. Western blot of cGAS-STING pathway.

Supplementary Figure 10. ICD induction.

Supplementary Figure 11. In situ tumor vaccination induced by aAGd-NWs sensitized radiation.

Supplementary Figure 12. The in vivo immunostimulation of APCs by aAGd-NWs sensitized RT.

Supplementary Figure 13. The flow cytometry of mature dendritic cells.

Supplementary Figure 14. Photographs of isolated tumors.

Supplementary Figure 15. Growth curves of individual mouse.

Supplementary Figure 16. Tumor weights CT26-bearing mice.

Supplementary Figure 17. Systemic antitumor immunity induced by aAGd-NWs sensitized radiation.

Supplementary Figure 18. Therapeutics of 4T1 metastatic breast cancer.

Supplementary Figure 19. Degradation mechanism of aAGd-NWs.

Supplementary Figure 20. Acute toxicity of aAGd-NWs and free $GdCl_3$ in healthy BALB/c mice.

Supplementary Figure 21. Serum biochemical analysis.

Supplementary Figure 22. H&E staining.

Supplementary Figure 23. Synthesis and CD characterization of aAGd-NWs and AGd-NCPs.

Supplementary Figure 24. Calculation of AMP and ara-AMP.

Supplementary Table 1. Cryo-EM data collection, processing and refinement.

Supplementary Table 2. Pharmacokinetic parameters of ara-AMP.

Supplementary Movie 1. Supplementary Movie of aAGd-NWs.

Source data

Note: Supplementary Movie 1 and Source data are provided as separate files.

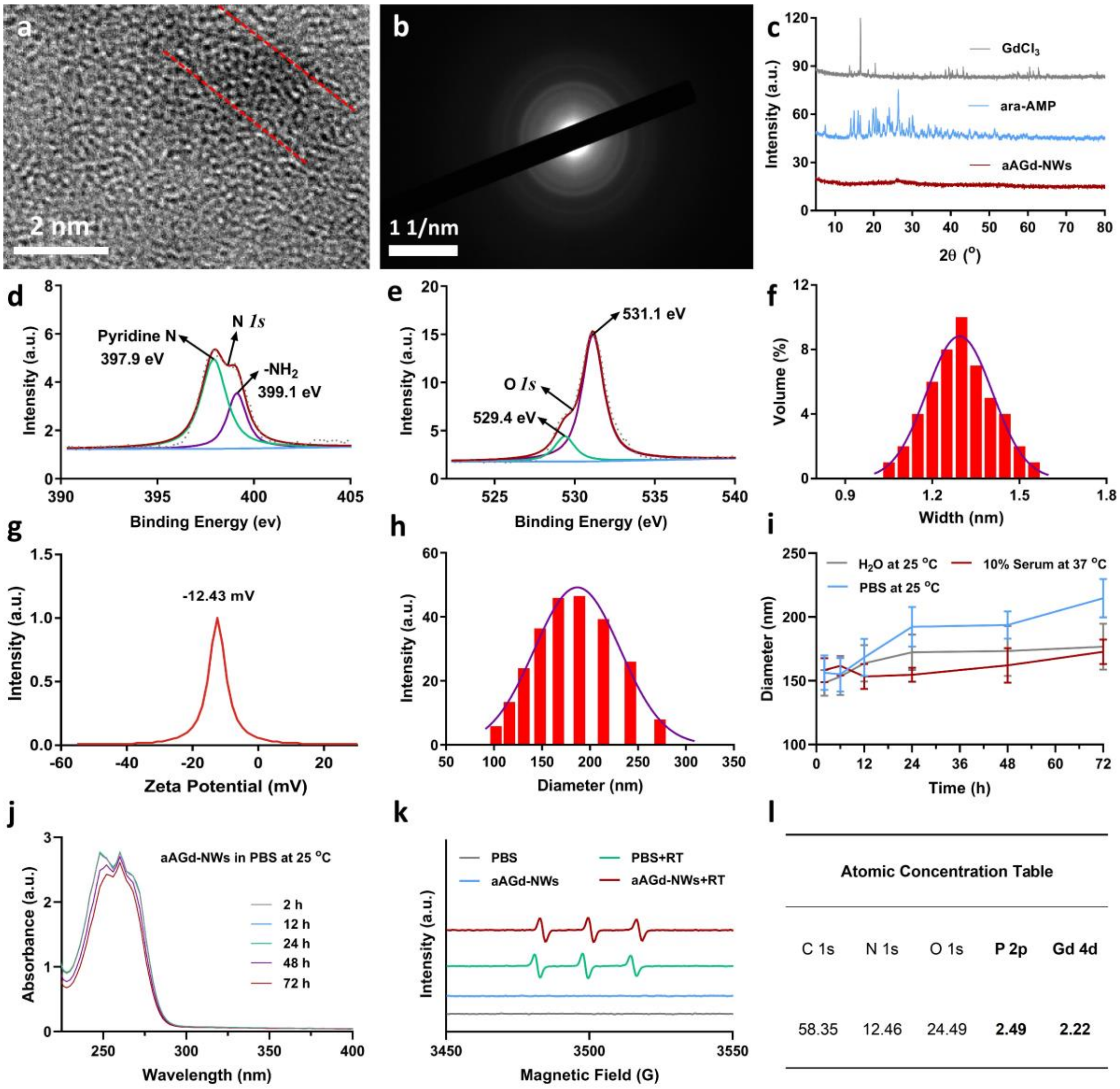

Supplementary Figure 1. Preparation and characterization of aAGd-NWs. (a) High resolution transmission electron microscope (HRTEM) imaging of aAGd-NWs, scale bar = 2 nm. This experiment was repeated twice independently with similar results. (b) Electron diffraction imaging of aAGd-NWs, scale bar = 1/nm. (c) Experimental powder X-ray diffraction (PXRD) patterns of $GdCl_3$, ara-AMP and aAGd-NWs. (d, e) High-resolution N *1s* (d) and O *1s* (e) XPS spectra of ara-AMP. (f) The width of the aAGd-NWs based on the HRTEM images. (g) Zeta potential of aAGd-NWs in deionized H_2O (n = 3 independent experiments). (h) Hydrated particle size of aAGd-NWs (n = 3

independent experiments). (i) DLS data of aAGd-NWs diluted with water (25 ^{o}C), PBS (25 ^{o}C) or 10% FBS (37 ^{o}C), respectively (n = 3 independent experiments). (j) Representative UV-vis spectra of aAGd-NWs in PBS (25 ^{o}C) at different time points (n = 3 independent experiments). (k) Electron Spin Resonance (ESR) spectra of singlet oxygen detection for different treatments. 2,2,6,6-Tetramethyl-4-piperidinone (TEMP) was used as a trap for singlet oxygen ($^{1}O_2$). (l) The atomic concentration table of the freeze-dried powder of non-dispersed ara-AMP-Gd coordination polymers. All experiments were repeated twice independently with similar results. Source data are provided as a Source Data file.

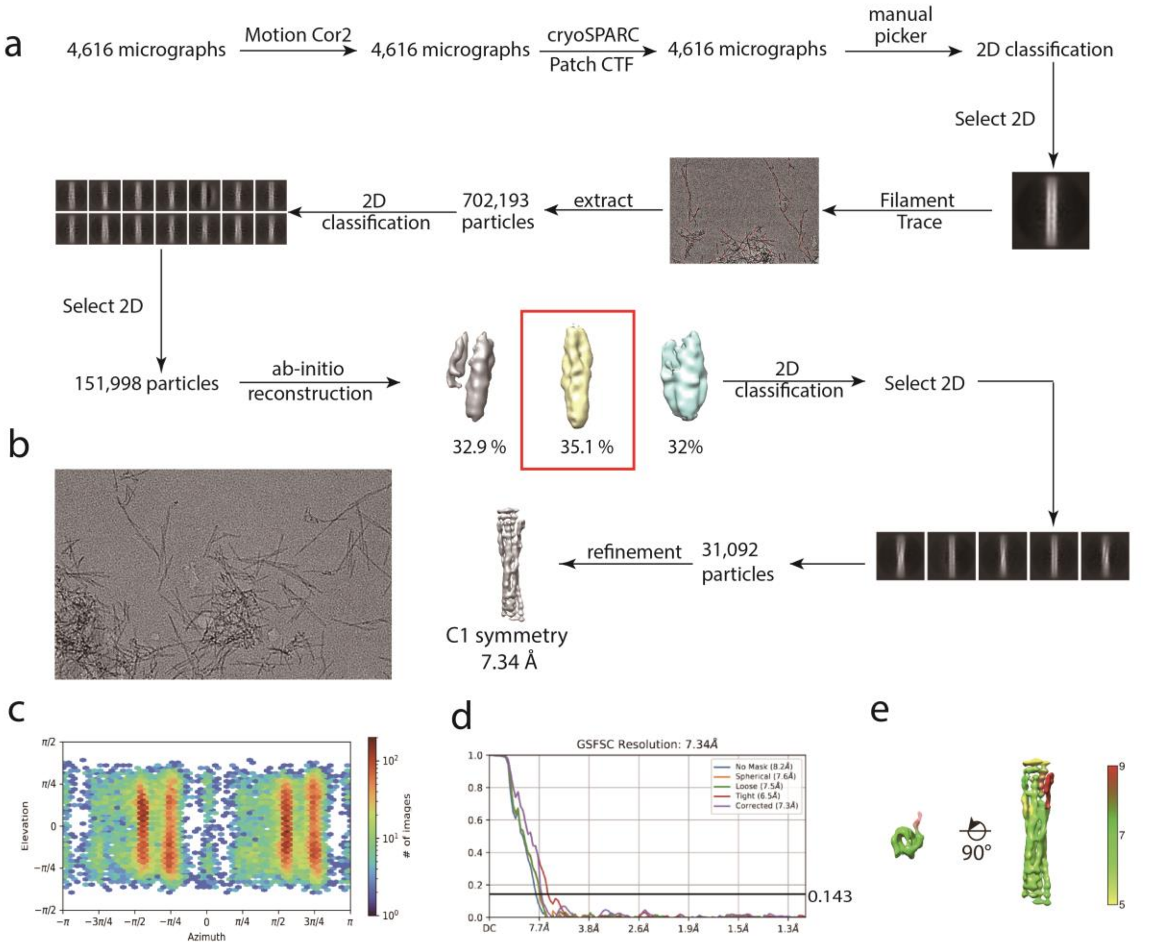

Supplementary Figure 2. Cryo-EM analysis of aAGd-NWs. (a) Summary of single-

particle image analysis procedure in cryoSPARC. (b) A raw image of aAGd-NWs. This experiment was repeated twice independently with similar results. (c) Particle orientation distribution. (d) FSC curve. (e) Local resolution map of aAGd-NWs.

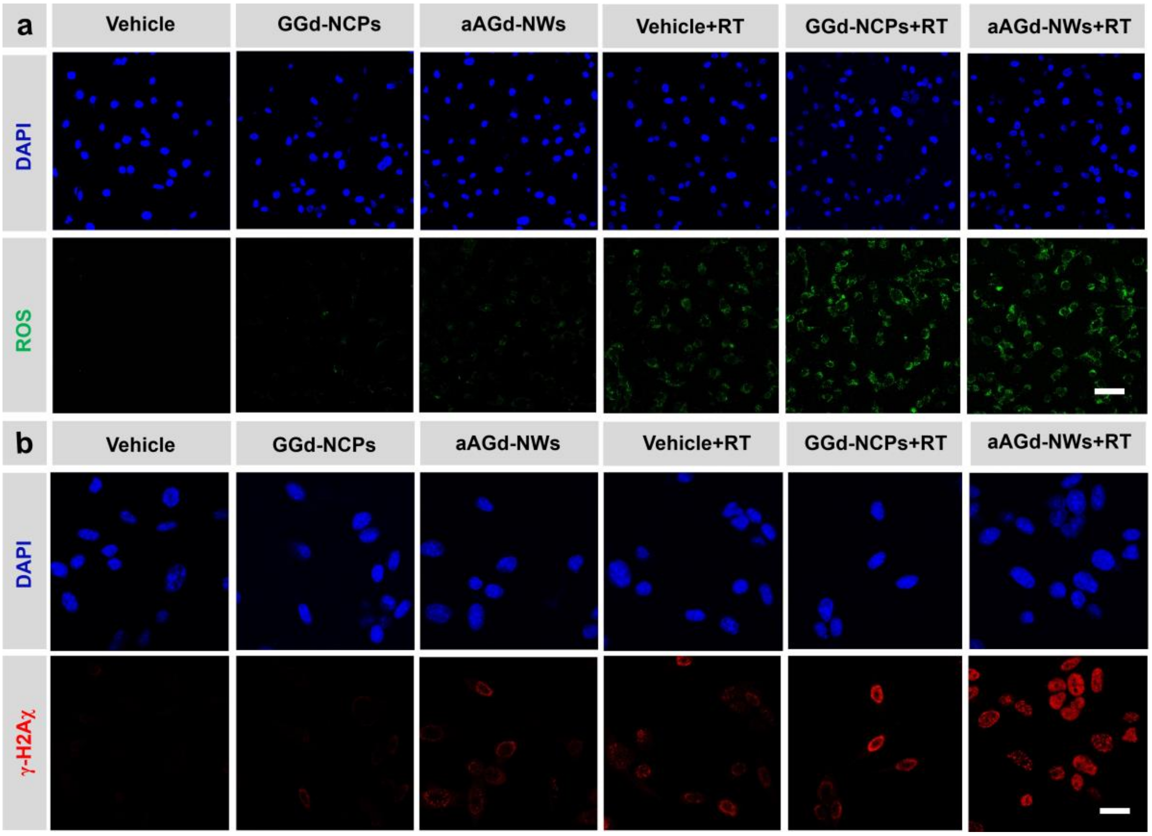

Supplementary Figure 3. Radiosensitization of aAGd-NWs in vitro. (a) Intracellular ROS generation in CT26 cells detected by ROS probe H_2DCFDA (green fluorescence) merged with DAPI (blue fluorescence), scale bar = 50 μm. (b) Intracellular γ-H2Aχ (red fluorescence) evaluation of CT26 cells, scale bar = 10 μm. All experiments were repeated twice independently with similar results.

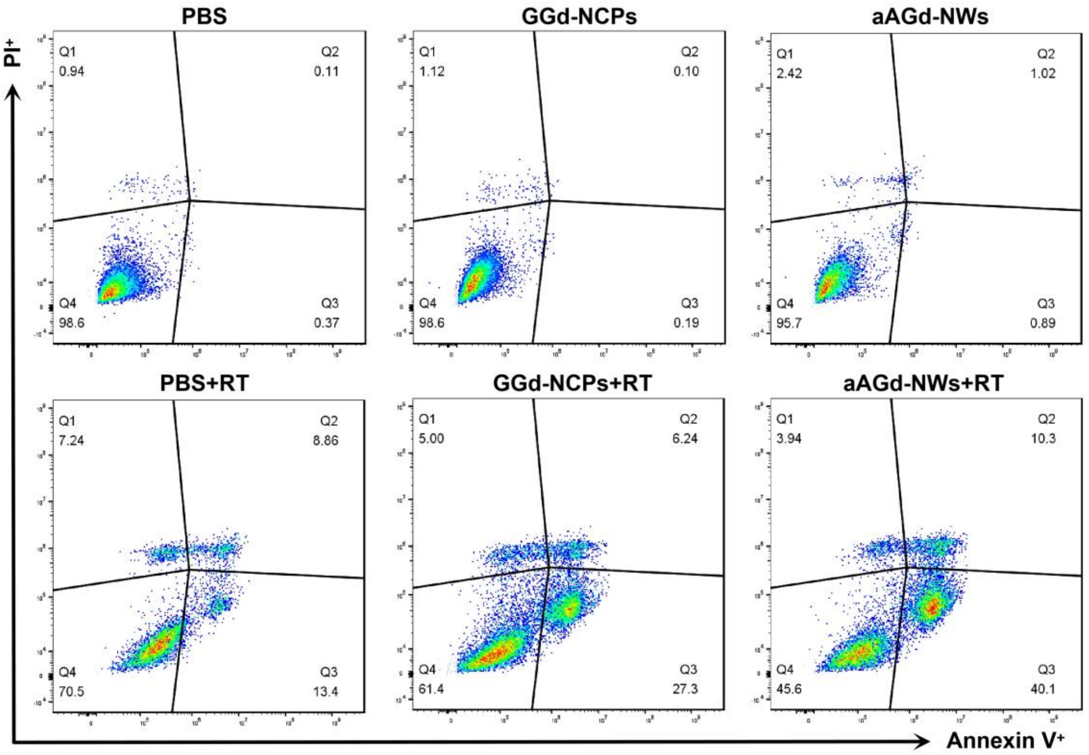

Supplementary Figure 4. Flow cytometry analysis of apoptosis. Flow cytometry analysis of apoptosis status of treated CT26 cells (n = 3 experimental repeats).

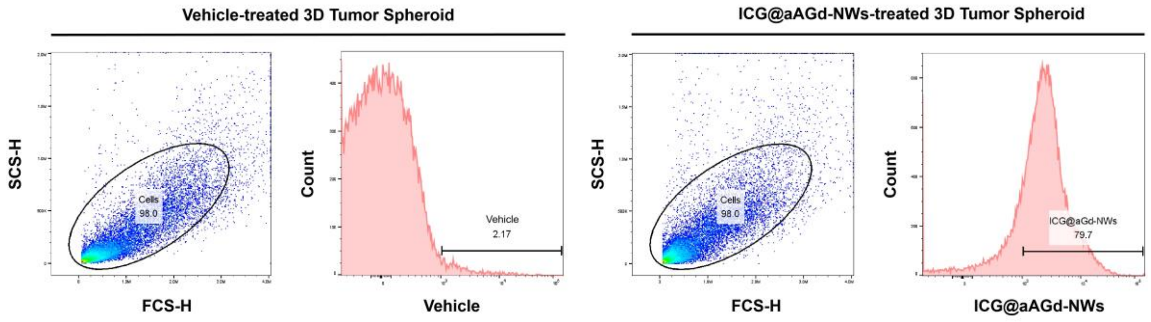

Supplementary Figure 5. Flow cytometry analysis of 3D tumor spheroids. Flow cytometry analysis of the dissociated tumor cells from spheroids treated by Vehicle or ICG@aAGd-NWs for 24 hours. This experiment was repeated twice independently with similar results.

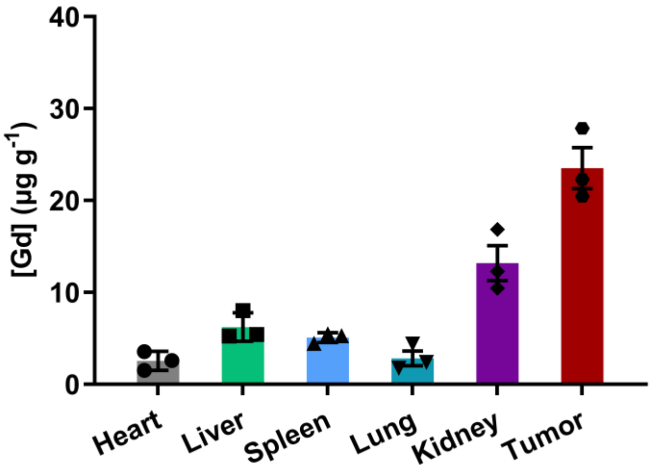

Supplementary Figure 6. The biodistribution of aAGd-NWs. The biodistribution of aAGd-NWs in tumors and major organs detected by ICP-OES (n = 3 mice). Source data are provided as a Source Data file.

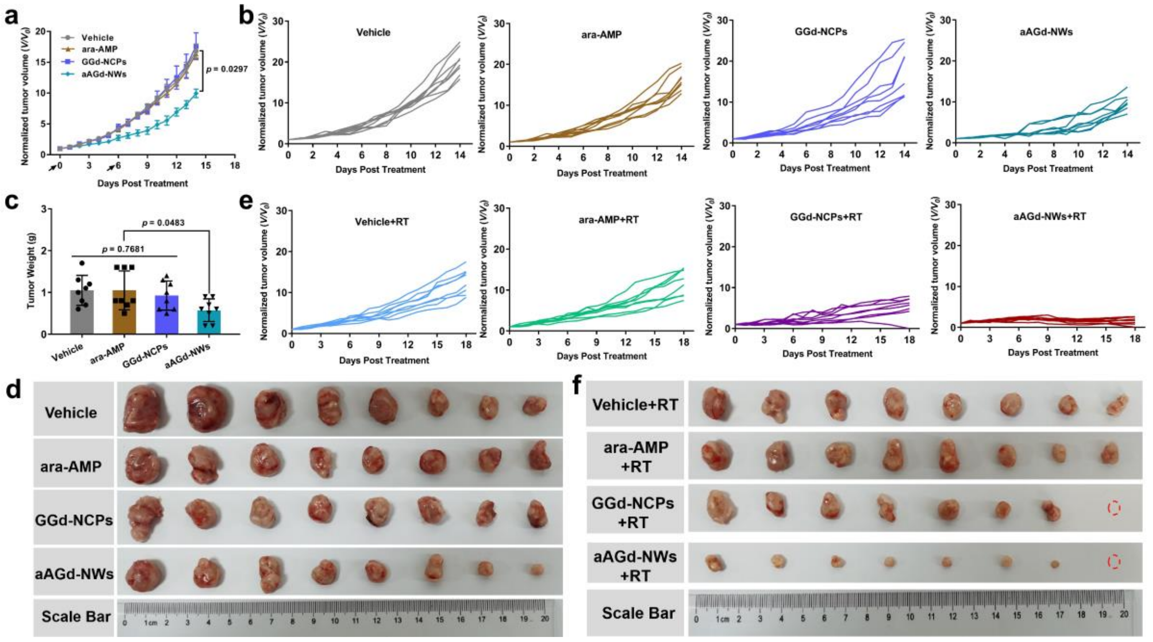

Supplementary Figure 7. Radiosensitization efficacy evaluation in vivo. (a) Normalized tumor growth curves (V/V_0) of Vehicle, free ara-AMP, GGd-NCPs, and aAGd-NWs treatments without X-ray irradiation (n = 8 mice). (b) Normalized growth

curves of individual tumor in Vehicle, free ara-AMP, GGd-NCPs, and aAGd-NWs groups. (c) Tumor weights after Vehicle, free ara-AMP, GGd-NCPs, and aAGd-NWs treatments without irradiation (n = 8 mice). (d) Photographs of tumors collected from Vehicle, free ara-AMP, GGd-NCPs, and aAGd-NWs groups on day 14. (e) Normalized growth curves of individual tumor in Vehicle+RT, free ara-AMP+RT, GGd-NCPs+RT, and aAGd-NWs+RT groups. (f) Photographs of tumors collected from Vehicle+RT, free ara-AMP+RT, GGd-NCPs+RT, and aAGd-NWs+RT groups on day 18. Source data are provided as a Source Data file.

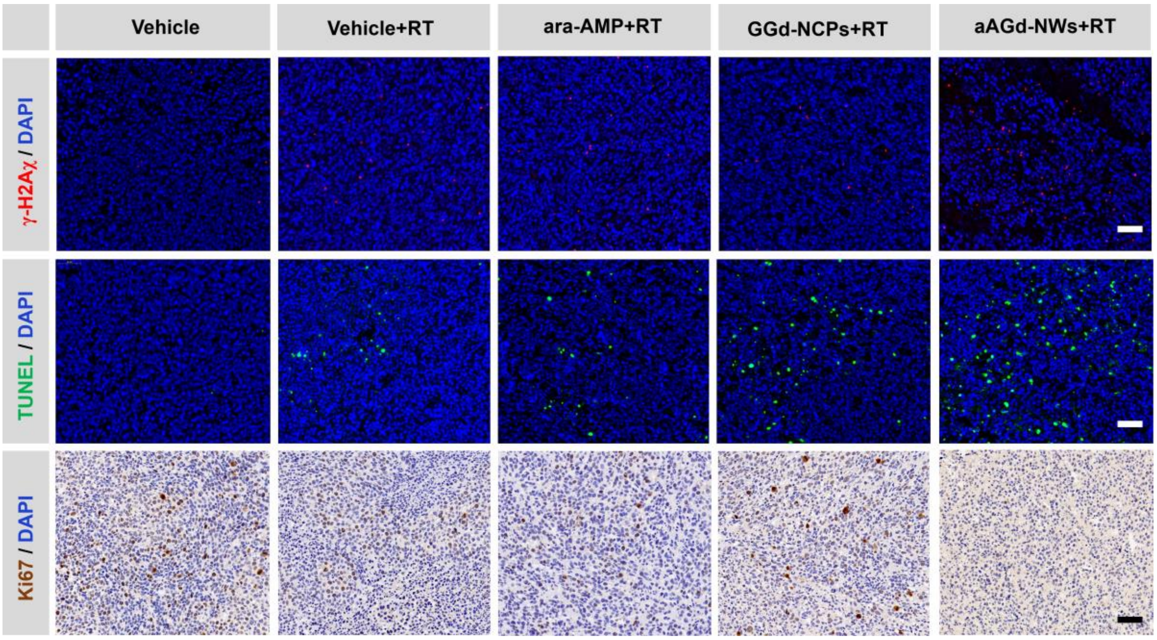

Supplementary Figure 8. Immunofluorescence and immunohistochemical staining. Tumor slices stained with γ-H2Aχ antibody, TUNEL and Ki67, scale bar = 50 μm. This experiment was repeated twice independently with similar results.

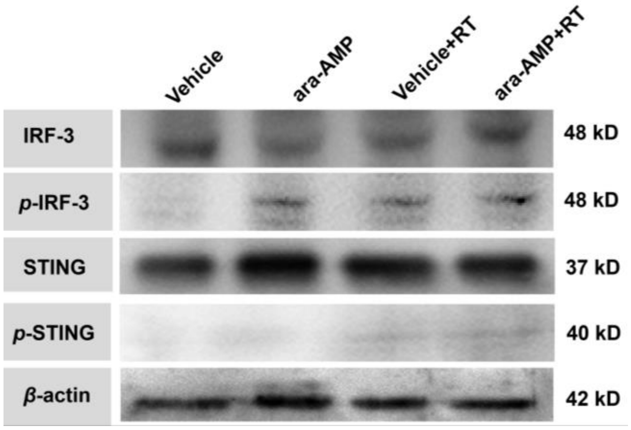

Supplementary Figure 9. Western blot of cGAS-STING pathway. Western blot of Vehicle, ara-AMP, Vehicle+RT and ara-AMP+RT groups. This experiment was repeated twice independently with similar results. Source data are provided as a Source Data file.

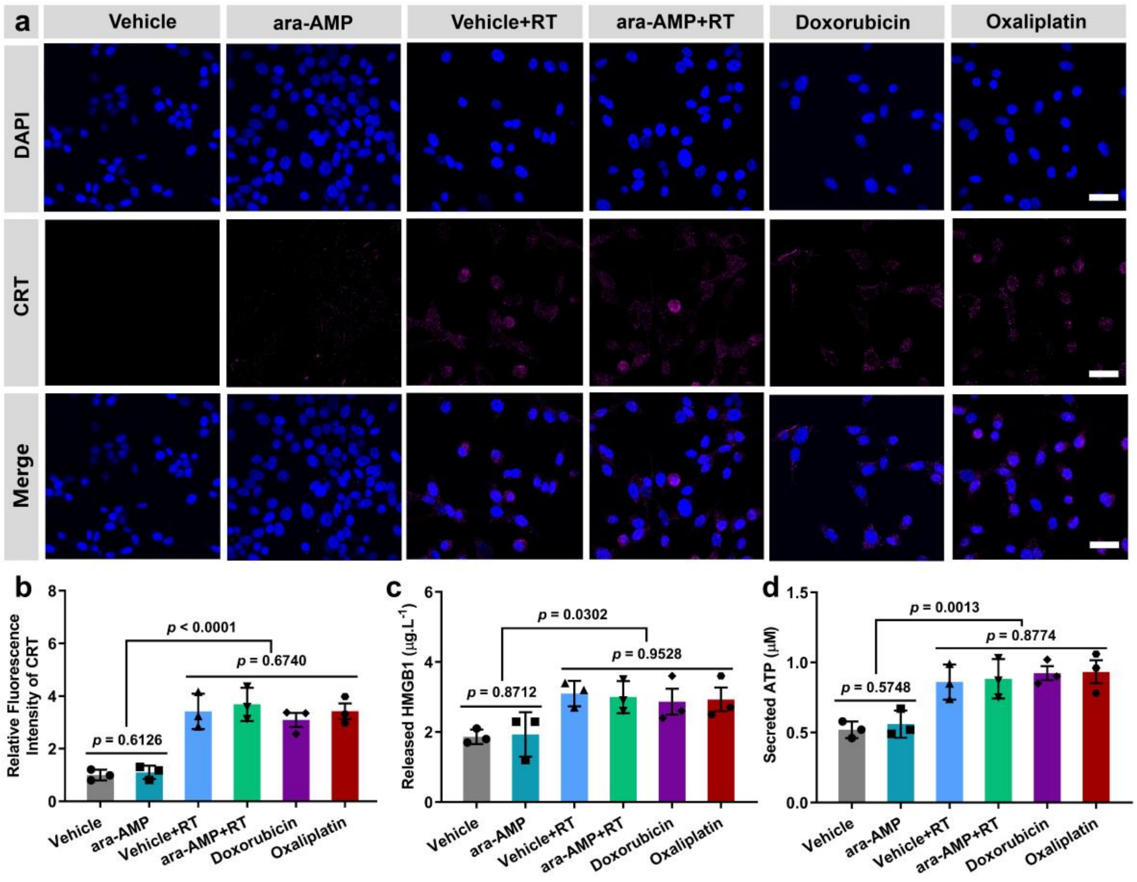

Supplementary Figure 10. ICD induction. (a) Immunofluorescence of CT26 cells

stained with anti-CRT antibody, scale bar = 50 μm. This experiment was repeated twice independently with similar results. (b) Quantification of relative CRT mean fluorescent intensity (n = 3 experimental repeats). (c, d) Detection of cytoplasmic HMGB1 (c) and ATP secretion (d) by ELISA kit and luciferin-based ATP assay kit (n = 3 experimental repeats). All data were shown as mean ± SD. Two-tailed Student's t-test was used to calculate statistical differences between two groups, and one-way ANOVA analysis of variance was used for multiple groups. p values > 0.05 represented nonsignificance (N.S.) and p values < 0.05 represented statistically significant. Source data are provided as a Source Data file.

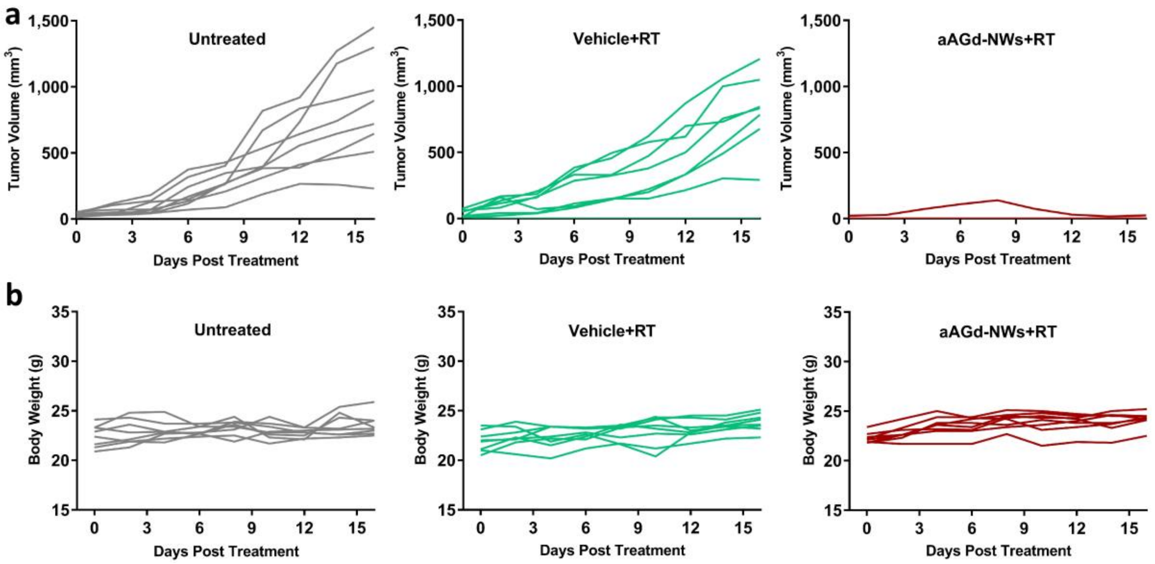

Supplementary Figure 11. In situ tumor vaccination induced by aAGd-NWs sensitized radiation. (a) Growth curves of individual mouse after treatments in Untreated, Vehicle+RT, aAGd-NWs+RT groups. (b) Body changes of individual mouse after treatments in Untreated, Vehicle+RT, aAGd-NWs+RT groups. Source data are provided as a Source Data file.

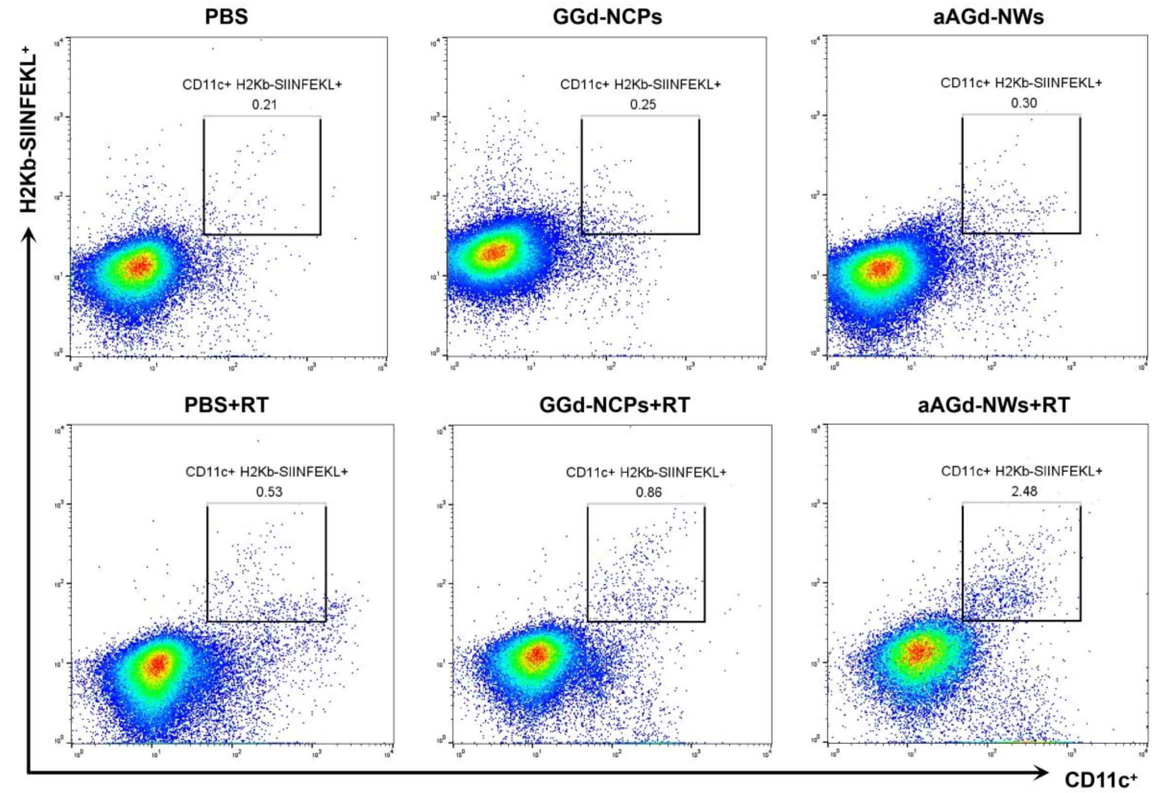

Supplementary Figure 12. The in vivo immunostimulation of APCs by aAGd-NWs sensitized RT. Flow cytometry imaging of H2Kb-SIINFEKL^{+} CD11c^{+} DCs in tumor draining lymph nodes after Vehicle, ara-AMP, GGd-NCPs, aAGd-NWs, Vehicle+RT, ara-AMP+RT, GGd-NCPs+RT, aAGd-NWs+RT treatments (n = 3 mice).

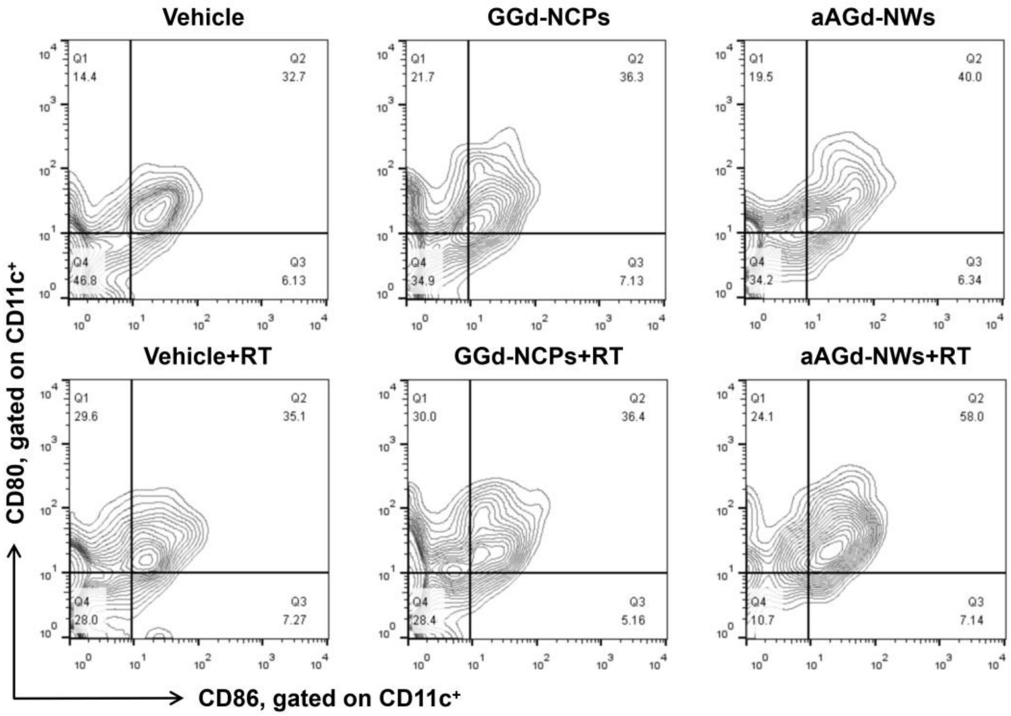

Supplementary Figure 13. The flow cytometry of mature dendritic cells. $CD80^+$ and $CD86^+$ gated within $CD11c^+$ cells in tumor-draining lymph nodes detected by flow cytometry (n = 6 mice).

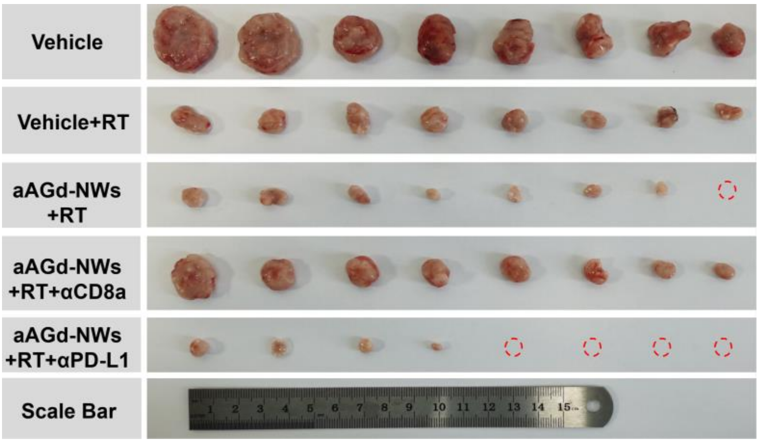

Supplementary Figure 14. Photographs of isolated tumors. Photographs of tumors of individual mouse after treatments in Vehicle, Vehicle+RT, aAGd-NWs+RT, aAGd-NWs +RT+αCD8a and aAGd-NWs+RT+αPD-L1 groups.

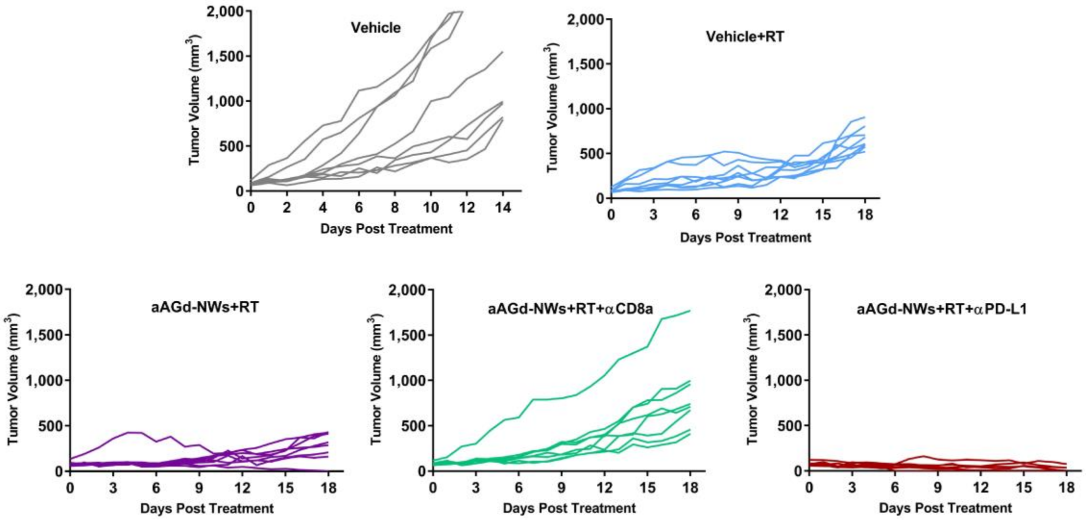

Supplementary Figure 15. Growth curves of individual mouse. Growth curves of individual mouse after treatments in Vehicle, Vehicle+RT, aAGd-NWs+RT, aAGd-NWs+RT+αCD8a and aAGd-NWs+RT+αPD-L1 groups. Source data are provided as a Source Data file.

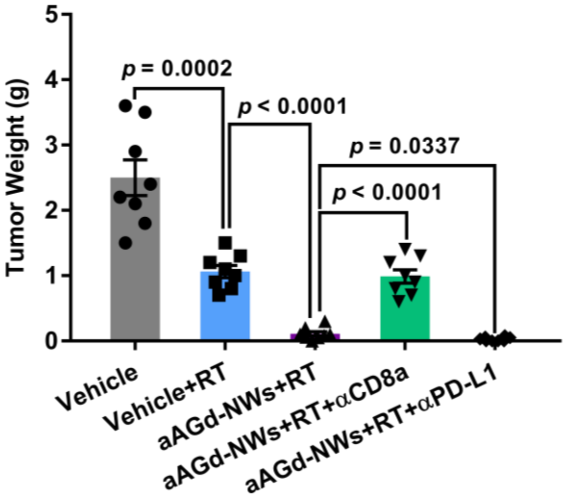

Supplementary Figure 16. Tumor weights CT26-bearing mice. Tumor weights of CT26-bearing mice after treatments in Vehicle, Vehicle+RT, aAGd-NWs+RT, aAGd-NWs+RT+αCD8a and aAGd-NWs+RT+ αPD-L1 groups (n = 8 mice). Source data are provided as a Source Data file.

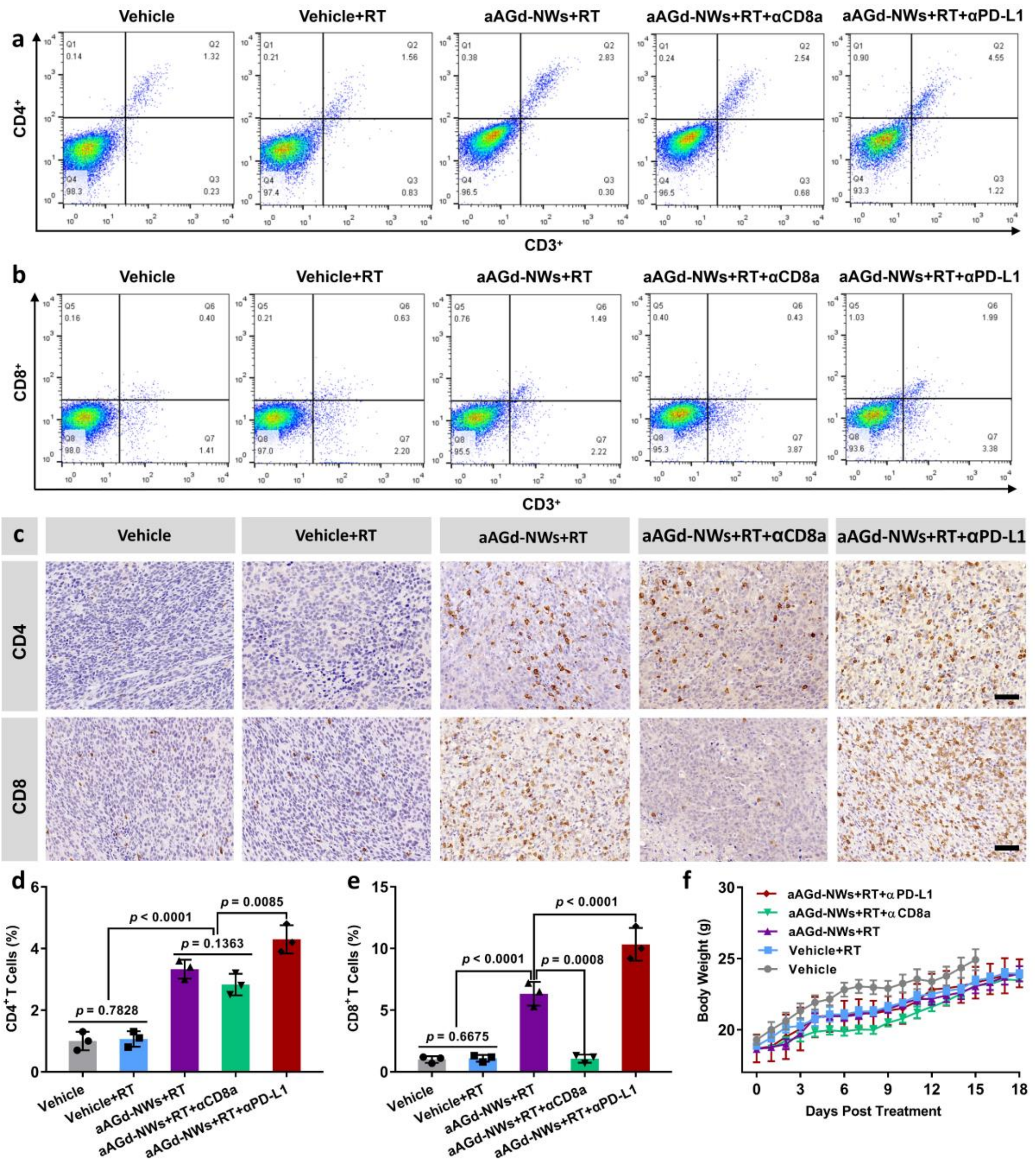

Supplementary Figure 17. Systemic antitumor immunity induced by aAGd-NWs sensitized radiation. (a, b) CD3^{+} CD4^{+} T cells (a) and CD3^{+} CD8^{+} T cells (b) detected by flow cytometry with Vehicle, Vehicle+RT, aAGd-NWs+RT, aAGd-NWs+RT+αCD8a and aAGd-NWs+RT+αPD-L1 treatments in CT26-bearing mice, respectively. (c) Immumohisto-chemical (IHC) staining of CD4^{+} and CD8^{+} T cells infiltrated in tumor

tissues, scale bar = 50 μm (n = 3 mice). This experiment was repeated twice independently with similar results. (d, e) Quantification of $CD4^+$ T cells (d) and $CD8^+$ T cells (e) infiltrated in tumor tissues based on IHC (n = 3 mice). (f) Dynamic body weights of CT26-bearing mice (n = 8 mice). All data were shown as mean ± SD. Two-tailed Student's t-test was used to calculate statistical differences between two groups, and one-way ANOVA analysis of variance was used for multiple groups. p values > 0.05 represented nonsignificance (N.S.) and p values < 0.05 represented statistically significant. Source data are provided as a Source Data file.

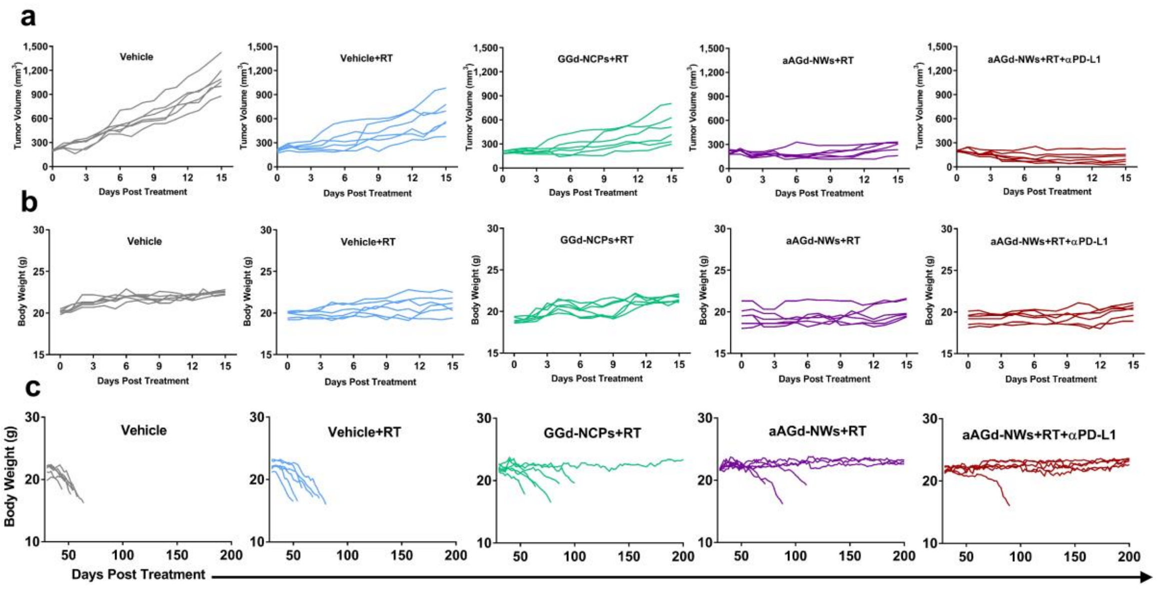

Supplementary Figure 18. Therapeutics of 4T1 metastatic breast cancer. (a) Growth curves of individual 4T1 tumor after treatments in Vehicle, Vehicle+RT, GGd-NCPs+RT, aAGd-NWs+RT and aAGd-NWs+RT+αPD-L1 groups. (b) Body changes of individual mouse after various treatments. (c) Body weight change profiles of treated 4T1 breast tumor-bearing mice during a 200-day observation period. Source data are provided as a Source Data file.

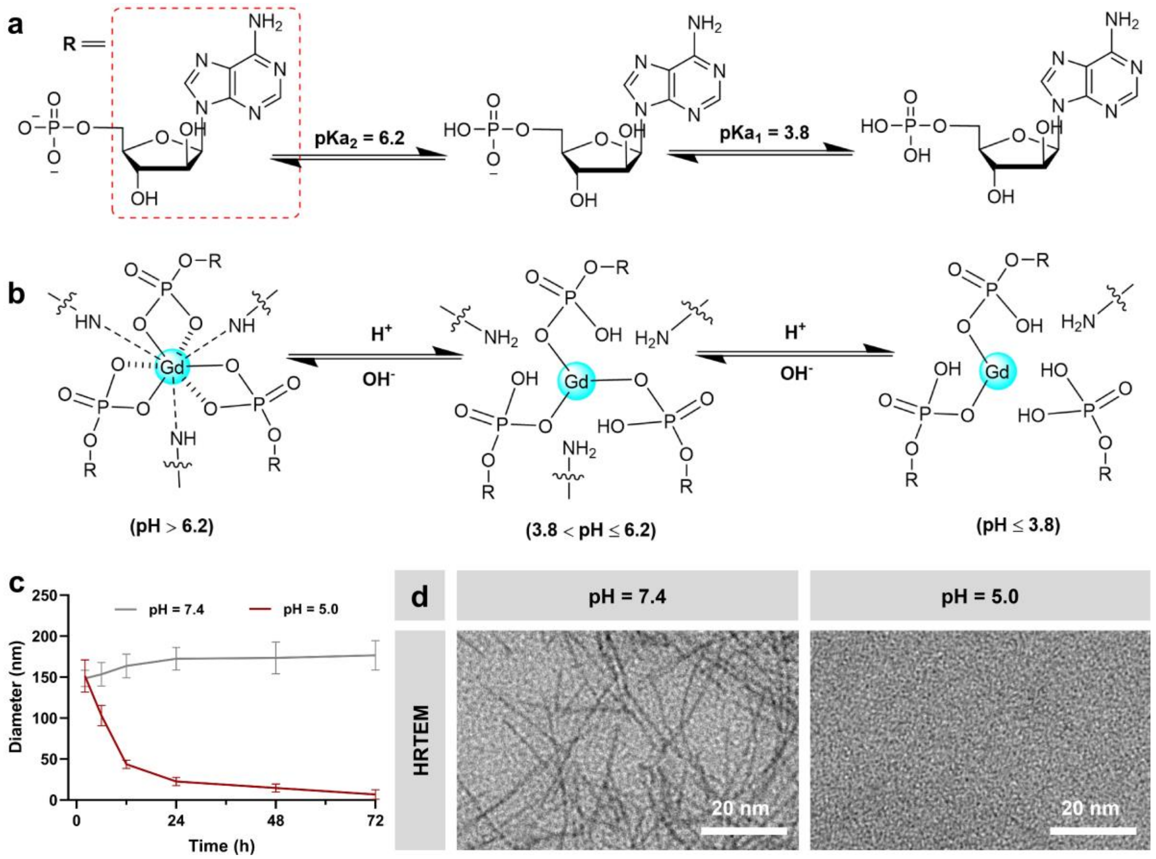

Supplementary Figure 19. Degradation mechanism of aAGd-NWs. (a) Schematic illustration of ara-AMP secondary acid dissociation. (b) Proposed mechanism of the pH dependent degradation process of aAGd-NWs. (c) DLS data of aAGd-NWs in HEPES buffer containing 10% serum at different pH values (7.4, 5.0) in vitro (n = 3 independent experiments). (d) High-resolution transmission electron microscopy (HRTEM) images of aAGd-NWs incubated for 48 hours under different pH values (7.4, 5.0) in vitro. This experiment was repeated twice independently with similar results. Source data are provided as a Source Data file.

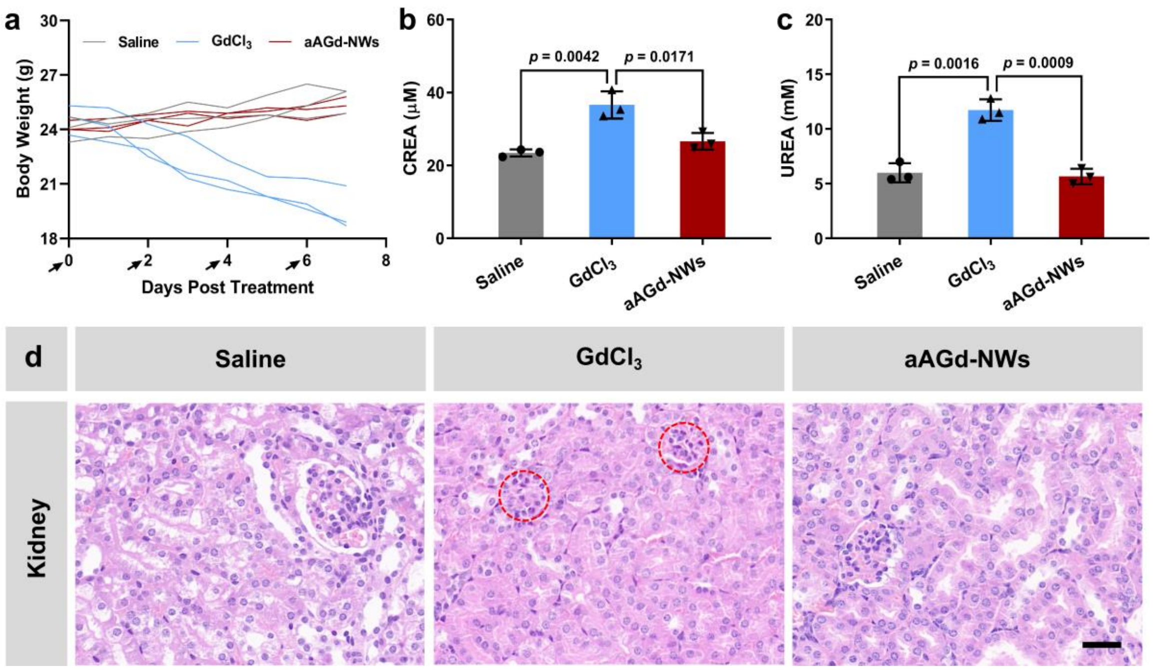

Supplementary Figure 20. Acute toxicity of aAGd-NWs and free $GdCl_3$ in healthy BALB/c mice. (a) Body weight change curves of individual mouse after different treatments. (b) Creatinine (CREA) and (c) urea (UREA) of CT26-bearing mice treated with Saline, free $GdCl_3$ and aAGd-NWs (n = 3 mice). (d) H&E stain sections of kidneys treated with Saline, free $GdCl_3$ and aAGd-NWs, scale bar = 20 μm. This experiment was repeated twice independently with similar results. All data were shown as mean ± SD. Source data are provided as a Source Data file.

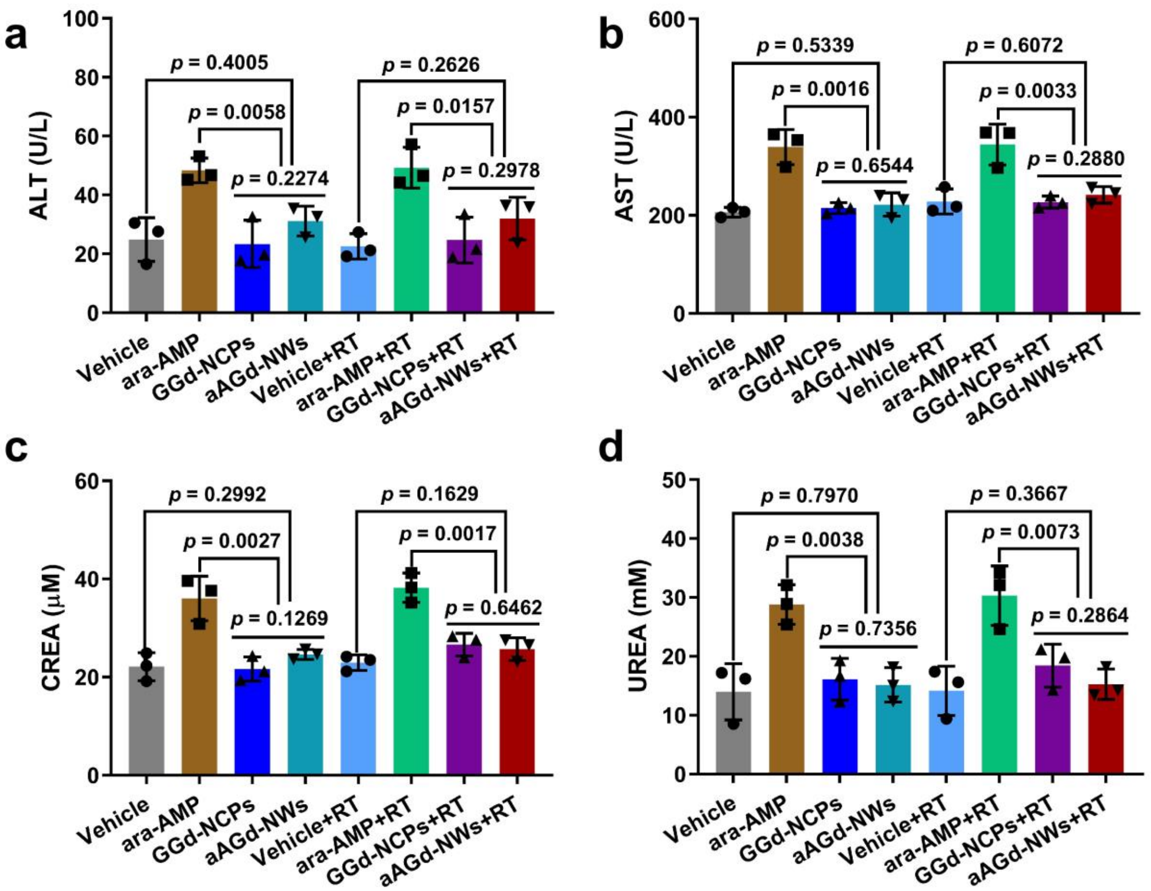

Supplementary Figure 21. Serum biochemical analysis. (a) Alanine aminotransferase (ALT), (b) aspartate aminotransferase (AST), (c) CREA and (d) UREA of CT26-bearing mice after various treatments (n = 3 mice). All data were shown as mean ± SD. Statistical significance was determined using two-tailed Student's t-test for pairwise comparisons, and one-way ANOVA analysis of variance for multiple groups. p values > 0.05 were considered non-significant (N.S.), while p values < 0.05 were considered statistically significant. Source data are provided as a Source Data file.

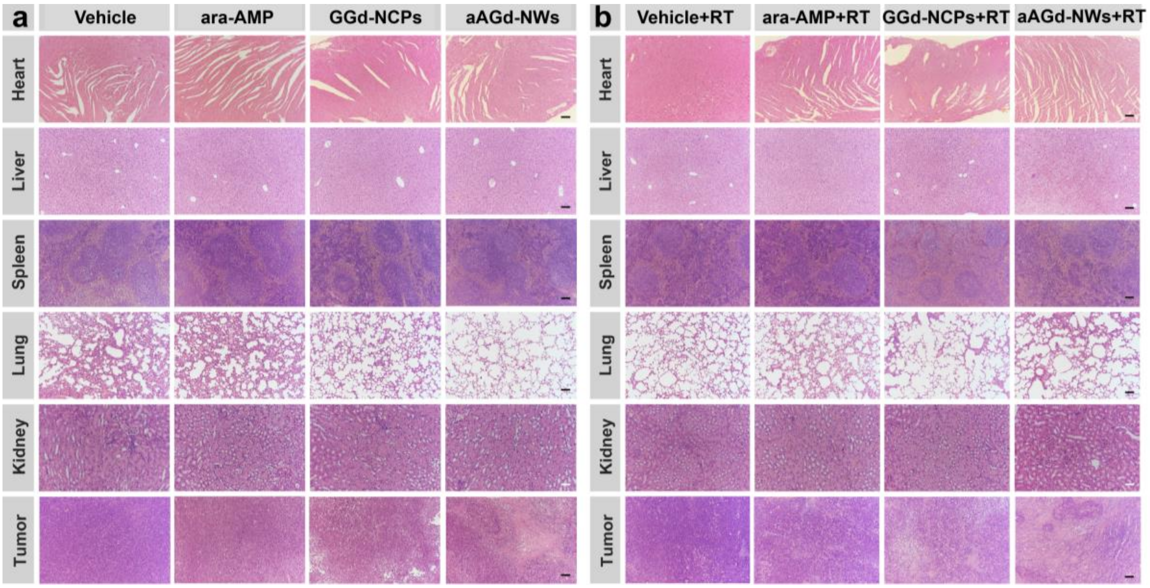

Supplementary Figure 22. H&E staining. (a, b) H&E stain sections of Heart, Liver, Spleen, Lung, Tumor (scale bar = 100 μm), and Kidney (scale bar = 50 μm) in Vehicle, free ara-AMP, GGd-NCPs, and aAGd-NWs treatments without (a) or with (b) X-ray irradiation groups (n = 3 mice). This experiment was repeated twice independently with similar results.

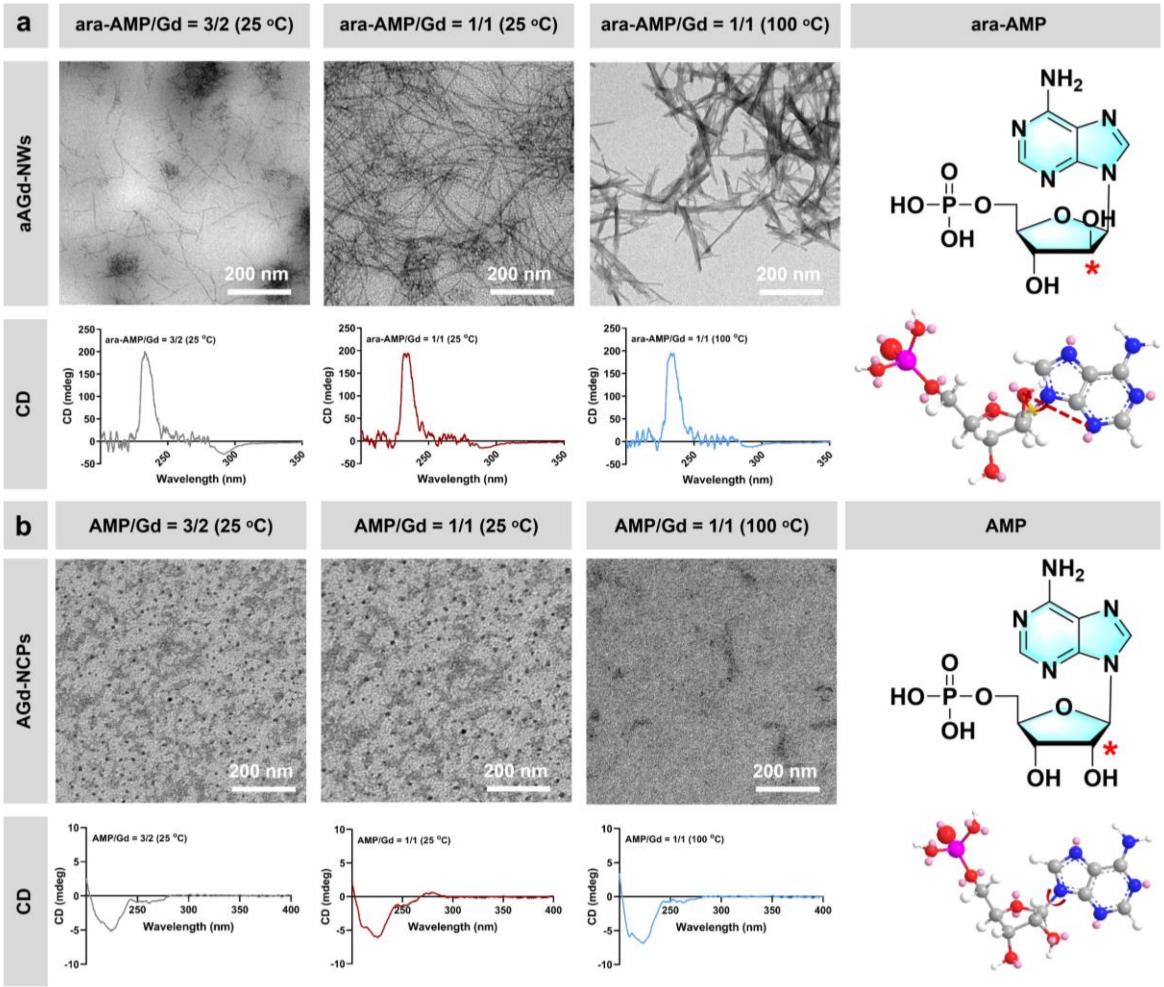

Supplementary Figure 23. Synthesis and CD characterization of aAGd-NWs and AGd-NCPs. (a) Synthesis and CD characterization of aAGd-NWs. (b) Synthesis and CD characterization of AGd-NCPs. All experiments were repeated twice independently with similar results. Source data are provided as a Source Data file.

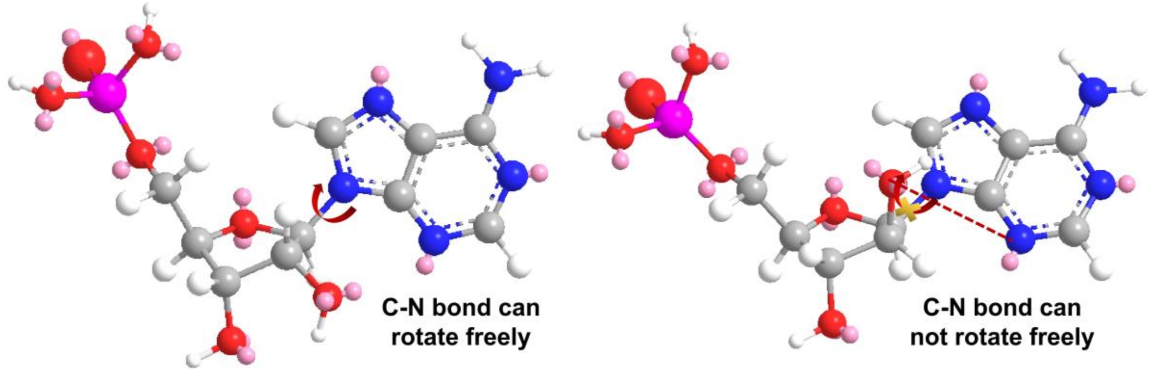

AMP	
Stretch:	1.2787
Bend:	27.5086
Stretch-Bend:	-0.1370
Torsion:	33.7528
Non-1,4 VDW:	-8.4852
1,4 VDW:	12.7742
Dipole/Dipole:	-8.6275
Total Energy:	58.0646 kcal mol^{-1}

ara-AMP	
Stretch:	1.2088
Bend:	27.2530
Stretch-Bend:	-0.1378
Torsion:	32.9977
Non-1,4 VDW:	-9.3171
1,4 VDW:	12.7741
Dipole/Dipole:	-8.2689
Total Energy:	56.5097 kcal mol^{-1}

Supplementary Figure 24. Calculation of AMP and ara-AMP. Calculate the minimum value of the conformational energy (MM2) of AMP and ara-AMP by Chem 3D. This calculation was repeated twice independently with similar results.

Supplementary Table 1. Cryo-EM data collection, processing and refinement.

**Data collection and processing**	
Magnification	81,000
Voltage (kV)	300
Electron exposure (e–/Å2)	50
Defocus range (μm)	-1.3~-1.8
Pixel size (Å)	0.6
Symmetry imposed	C1
Initial particle images (no.)	702,193
Final particle images (no.)	31,092
Map resolution (Å)	7.34
FSC threshold	0.143
Map resolution range (Å)	5~9
**Refinement**	
Map sharpening B factor (Å2)	827.8

Supplementary Table 2. Pharmacokinetic parameters of ara-AMP*.

**Pharmacokinetic Parameters**	**AUC_{0-t}** (mg L^{-1}*h)	**$AUMC_{0-t}$** (mg L^{-1}*h^2)	**MRT_{0-t}** (h)	**$t_{1/2}$** (h)	**C_{max}** (mg L^{-1})
Free ara-AMP	350±55.16	1183±98.11	3.38±0.23	3.52±0.46	96.6±11.5
aAGd-NWs	1075±98.87	7578±389.55	7.05±0.38	7.28±0.55	102.6±10.8

*Data was shown as mean ± SD (n = 3 experimental repeats). The pharmacokinetic parameters of ara-AMP were analyzed by DAS 2.1.1 software. Source data are provided as a Source Data file.
