# Supplementary material for: Chiral coordination polymer nanowires boost radiation-induced in situ tumor vaccination
Source: Nat Commun. 2024 May 9;15:3902. doi: 10.1038/s41467-024-48423-w (PMC11082158; doi:10.1038/s41467-024-48423-w)
Supplement: Supplementary file 3 — Description of Additional Supplementary Files [file 41467_2024_48423_MOESM3_ESM.pdf]

## Description of Additional Supplementary Files

**Supplementary Movie 1:** Supplementary Movie of aAGd-NWs.
