# Supplementary material for: Chiral coordination polymer nanowires boost radiation-induced in situ tumor vaccination
Source: Nat Commun. 2024 May 9;15:3902. doi: 10.1038/s41467-024-48423-w (PMC11082158; doi:10.1038/s41467-024-48423-w)
Supplement: Supplementary file 5 — Reporting Summary [file 41467_2024_48423_MOESM5_ESM.pdf]

nature portfolio

Corresponding author(s): Zhimin Luo, Yiqiao Hu, Ahu Yuan

Last updated by author(s): 18/03/2024

Reporting Summary

Nature Portfolio wishes to improve the reproducibility of the work that we publish. This form provides structure for consistency and transparency in reporting. For further information on Nature Portfolio policies, see our Editorial Policies and the Editorial Policy Checklist.

Statistics

For all statistical analyses, confirm that the following items are present in the figure legend, table legend, main text, or Methods section.

n/a	Confirmed	
☐	☒	The exact sample size (*n*) for each experimental group/condition, given as a discrete number and unit of measurement
☐	☒	A statement on whether measurements were taken from distinct samples or whether the same sample was measured repeatedly
☐	☒	The statistical test(s) used AND whether they are one- or two-sided<br>*Only common tests should be described solely by name; describe more complex techniques in the Methods section.*
☒	☐	A description of all covariates tested
☒	☐	A description of any assumptions or corrections, such as tests of normality and adjustment for multiple comparisons
☐	☒	A full description of the statistical parameters including central tendency (e.g. means) or other basic estimates (e.g. regression coefficient) AND variation (e.g. standard deviation) or associated estimates of uncertainty (e.g. confidence intervals)
☐	☒	For null hypothesis testing, the test statistic (e.g. *F*, *t*, *r*) with confidence intervals, effect sizes, degrees of freedom and *P* value noted<br>*Give P values as exact values whenever suitable.*
☒	☐	For Bayesian analysis, information on the choice of priors and Markov chain Monte Carlo settings
☒	☐	For hierarchical and complex designs, identification of the appropriate level for tests and full reporting of outcomes
☐	☒	Estimates of effect sizes (e.g. Cohen's *d*, Pearson's *r*), indicating how they were calculated

Our web collection on statistics for biologists contains articles on many of the points above.

Software and code

Policy information about availability of computer code

Data collection	The morphologies, crystal structures and elemental composition were collected using transmission electron microscopy (TEM) (Hitachi HT7700, Japan) and high-resolution TEM (HRTEM) combined energy dispersive spectroscopy (EDS) (JEM-2100F, JEOL, Japan). Cryo-Transmission Electron Microscope (cryo-TEM) images were acquired by the Thermo Scientific Glacios 2 cryo-EM (USA). Powder X-ray diffraction (PXRD) patterns were obtained using a D8 ADVANCE XRD (Bruker, Germany) with Cu Kα radiation (λ = 1.54 Å). X-ray photoelectron spectroscopy (XPS) spectra were collected using a PHI 5000 VersaProbe (Ulvac-Phi, Japan) with Al Kα radiation (hv = 1486.6 eV). Ultraviolet-visible (UV-vis) absorption spectra were recorded with a Shimadzu UV-vis spectrophotometer (UV3600, Japan). Fourier transform infrared (FT-IR) spectra were recorded with a Fourier-Transform Infrared Spectrometer (Bruker, Vertex 80v and Tensor 27, Germany). Electron Spin Resonance (ESR) specta were collected by the EMS Plus ESR Spectrometer (Bruker, Germany). The amount of Gd3+ was collected by an Inductively Coupled Plasma Optical Emission Spectrometer (ICP-OES) (Perkin Elmer Optima 5300 DV, USA). Flow cytometry for typing of all immune cells (BD, FACSCalibur, USA). The X-ray irradiation equipment is the Rad Source RS2000 X-ray irradiator (Model: X-ray Irradiator RS2000, USA). The instrument's technical specifications comprise a dose rate of 10 Gy min-1 for cells and 1.2 Gy min-1 for small animals. The irradiation plane is situated 25 cm away in the cone-shaped irradiation field. The operating specifications of the power supply are 220 volts, 60 Hz, 40 amps. Tumor size and weight of tumor-bearing mice were recorded with Microsoft Office 2019.
Data analysis	GraphPad Prism Version 9.0.2 was used to analyze statistical data. FlowJo (Version 10.8.1) was used to analyze flow cytometry data. CaseViewer Version 2.4 and ImageJ Version 1.52v were used to analyze immunofluorescent and immunochemical data. The pharmacokinetic parameters of ara-AMP were analyzed by DAS 2.1.1 software. Adobe Illustrator 2020 was used to create Fig. 1 and Fig 2b. BioRender.com was used to create Fig. 2a, 6a, 8a and 9c. Calculate the minimum value of the conformational energy (MM2) of AMP and ara-AMP by Chem 3D 20.0.

For manuscripts utilizing custom algorithms or software that are central to the research but not yet described in published literature, software must be made available to editors and reviewers. We strongly encourage code deposition in a community repository (e.g. GitHub). See the Nature Portfolio guidelines for submitting code & software for further information.

Data

Policy information about availability of data

All manuscripts must include a data availability statement. This statement should provide the following information, where applicable:
- Accession codes, unique identifiers, or web links for publicly available datasets
- A description of any restrictions on data availability
- For clinical datasets or third party data, please ensure that the statement adheres to our policy

The authors declare that data supporting the findings of this study are available within the article, and its Supplementary Information files and Source data are provided with this paper.

Research involving human participants, their data, or biological material

Policy information about studies with human participants or human data. See also policy information about sex, gender (identity/presentation), and sexual orientation and race, ethnicity and racism.

Reporting on sex and gender	This study did not involve human research participants.
Reporting on race, ethnicity, or other socially relevant groupings	This study did not involve human research participants.
Population characteristics	This study did not involve human research participants.
Recruitment	This study did not involve human research participants.
Ethics oversight	This study did not involve human research participants.

Note that full information on the approval of the study protocol must also be provided in the manuscript.

Field-specific reporting

Please select the one below that is the best fit for your research. If you are not sure, read the appropriate sections before making your selection.

☒ Life sciences ☐ Behavioural & social sciences ☐ Ecological, evolutionary & environmental sciences

For a reference copy of the document with all sections, see nature.com/documents/nr-reporting-summary-flat.pdf

Life sciences study design

All studies must disclose on these points even when the disclosure is negative.

Sample size	Sample sizes were assigned based upon historical practices, in line with our previous publications, Huang, Z., Wang, Y., Yao, D. et al. Nanoscale coordination polymers induce immunogenic cell death by amplifying radiation therapy mediated oxidative stress. Nat Commun 12, 145 (2021). https://doi.org/10.1038/s41467-020-20243-8.
Data exclusions	No data were excluded from analysis.
Replication	All experiments were replicated independently 2-3 times, as indicated in figure legends.
Randomization	Cell samples were randomly allocated to treatment by random division into multi-well plates. Animals were randomized by random number.
Blinding	Investigators were blinded to group allocation during data collection and analysis for both in vitro and in vivo experiments.

Reporting for specific materials, systems and methods

We require information from authors about some types of materials, experimental systems and methods used in many studies. Here, indicate whether each material, system or method listed is relevant to your study. If you are not sure if a list item applies to your research, read the appropriate section before selecting a response.

Materials & experimental systems

n/a	Involved in the study
☐	☒ Antibodies
☐	☒ Eukaryotic cell lines
☒	☐ Palaeontology and archaeology
☐	☒ Animals and other organisms
☒	☐ Clinical data
☒	☐ Dual use research of concern
☒	☐ Plants

Methods

n/a	Involved in the study
☒	☐ ChIP-seq
☐	☒ Flow cytometry
☒	☐ MRI-based neuroimaging

Antibodies

Antibodies used

1. Anti-gamma H2A.X (phospho S139) antibody [9F3], Cat# ab26350, diluted 1:200 with 3% BSA, Abcam;
2. TUNEL Assay Kit - BrdU-Red, Cat# ab66110, diluted 1:200 with 3% BSA, Abcam;
3. Anti-Ki67 antibody, Cat# ab15580, diluted 1:500 with 3% BSA, Abcam;
4. Anti-Calreticulin antibody [EPR3924] - ER Marker (Alexa Fluor® 488), Cat# ab196158, diluted 1:500 with 3% BSA, Abcam;
5. Anti-beta Actin antibody [mAbcam 8226] - Loading Control, Cat# ab8226, diluted 1:500 with 3% BSA, Abcam;
6. Recombinant Anti-STING antibody [EPR25090-107], Cat# ab288157, diluted 1:500 with 3% BSA, Abcam;
7. Recombinant Anti-IRF3 antibody [EPR2418Y], Cat# ab68481, diluted 1:500 with 3% BSA, Abcam;
8. Phospho-STING (Ser366) Polyclonal Antibody, Cat# PA5-105674, diluted 1:500 with 3% BSA, Thermofisher;
9. Phospho-IRF3 (Ser386) Polyclonal Antibody, Cat# PA5-121307, diluted 1:500 with 3% BSA, Thermofisher;
10. HRP conjugated Goat Anti-Rabbit IgG (H+L), Cat# GB23303, diluted 1:300 with 3% BSA, Servicebio;
11. APC anti-mouse CD80 Antibody [16-10A1], Cat# 104713, 1.0 μg per million cells in 100 μL volume, BioLegend;
12. PE anti-mouse CD86 Antibody [GL-1], Cat# 105007, 0.25 μg per million cells in 100 μL volume, BioLegend;
13. FITC anti-mouse CD11c Antibody [N418], Cat# 117306, 0.25 μg per million cells in 100 μL volume, BioLegend;
14. APC anti-mouse CD3 Antibody [17A2], Cat# 100236, 0.5 μg per million cells in 100 μL volume, BioLegend;
15. PE anti-mouse CD4 Antibody [GK1.5], Cat# 100408, 0.25 μg per million cells in 100 μL volume, BioLegend;
16. FITC anti-mouse CD8a Antibody [53-6.7], Cat# 100706, 1.0 μg per million cells in 100 μL volume, BioLegend;
17. APC anti-mouse H-2Kb Antibody [AF6-88.5], Cat# 116506, 1.0 μg per million cells in 100 μL volume, BioLegend;
18. Purified anti-mouse IFN-γ Antibody [R4-6A2], Cat# 505702, 2.0 μg mL-1, BioLegend;
19. Ultra-LEAF™ Purified anti-mouse CD8a Antibody [53-6.7], Cat# 100764, 10.0 mg kg-1, BioLegend;
20. In Vivo MAb anti-mouse PD-L1(B7-H1) [Clone: 10F.9G2], Cat# BE0101, 10.0 mg kg-1, BioXcell.

Validation

All antibodies were verified by the supplier and each lot has been quality tested.
1. Anti-gamma H2A.X (phospho S139) antibody [9F3], Cat# ab26350, diluted 1:200 with 3% BSA, Abcam, validated for immunofluorescence analysis and tested to detect in mouse by the manufacturer, https://www.abcam.com/gamma-h2ax-phospho-s139-antibody-9f3-ab26350.html.
2. TUNEL Assay Kit - BrdU-Red, Cat# ab66110, diluted 1:200 with 3% BSA, Abcam, validated for immunofluorescence analysis and tested to detect in mouse by the manufacturer, https://www.abcam.com/tunel-assay-kit-brdu-red-ab66110.html.
3. Anti-Ki67 antibody, Cat# ab15580, diluted 1:500 with 3% BSA, Abcam, validated for immunohistochemistry (IHC) analysis and tested to detect in mouse by the manufacturer, https://www.abcam.com/ki67-antibody-ab15580.html.
4. Anti-Calreticulin antibody [EPR3924] - ER Marker (Alexa Fluor® 488), Cat# ab196158, diluted 1:500 with 3% BSA, Abcam, validated for immunofluorescence analysis and tested to detect in mouse by the manufacturer, https://www.abcam.com/calreticulin-antibody-epr3924-er-marker-alexa-fluor-488-ab196158.html.
5. Anti-beta Actin antibody [mAbcam 8226] - Loading Control, Cat# ab8226, diluted 1:500 with 3% BSA, Abcam; validated for western blot (WB) and tested to detect in mouse by the manufacturer, https://www.abcam.com/products/primary-antibodies/beta-actin-antibody-mabcam-8226-loading-control-ab8226.html.
6. Recombinant Anti-STING antibody [EPR25090-107], Cat# ab288157, diluted 1:500 with 3% BSA, Abcam; validated for western blot (WB) and tested to detect in mouse by the manufacturer, https://www.abcam.com/products/primary-antibodies/sting-antibody-epr25090-107-ab288157.html.
7. Recombinant Anti-IRF3 antibody [EPR2418Y], Cat# ab68481, diluted 1:500 with 3% BSA, Abcam; validated for western blot (WB) and tested to detect in mouse by the manufacturer, https://www.abcam.com/products/primary-antibodies/irf3-antibody-epr2418y-ab68481.html.
8. Phospho-STING (Ser366) Polyclonal Antibody, Cat# PA5-105674, diluted 1:500 with 3% BSA, Thermofisher, validated for western blot (WB) and tested to detect in mouse by the manufacturer, https://www.thermofisher.com/antibody/product/Phospho-STING-Ser366-Antibody-Polyclonal/PA5-105674.
9. Phospho-IRF3 (Ser386) Polyclonal Antibody, Cat# PA5-121307, diluted 1:500 with 3% BSA, Thermofisher, validated for western blot (WB) and tested to detect in mouse by the manufacturer, https://www.thermofisher.com/antibody/product/Phospho-IRF3-Ser386-Antibody-Polyclonal/PA5-121307.
10. HRP conjugated Goat Anti-Rabbit IgG (H+L), Cat# GB23303, diluted 1:300 with 3% BSA, Servicebio, validated for immunohistochemistry (IHC) analysis and tested to detect in mouse by the manufacturer, https://www.servicebio.cn/goodsdetail?id=266.
11. APC anti-mouse CD80 Antibody [16-10A1], Cat# 104713, 1.0 μg per million cells in 100 μL volume, BioLegend, validated for flow cytometry and tested to detect in mouse by the manufacturer, https://www.biolegend.com/en-gb/products/apc-anti-mouse-cd80-antibody-2340.
12. PE anti-mouse CD86 Antibody [GL-1], Cat# 105007, 0.25 μg per million cells in 100 μL volume, BioLegend, validated for flow cytometry and tested to detect in mouse by the manufacturer, https://www.biolegend.com/en-gb/products/pe-anti-mouse-cd86-antibody-256.
13. FITC anti-mouse CD11c Antibody [N418], Cat# 117306, 0.25 μg per million cells in 100 μL volume, BioLegend, validated for flow cytometry and tested to detect in mouse by the manufacturer, https://www.biolegend.com/en-gb/products/fitc-anti-mouse-cd11c-

antibody-1815.
14. APC anti-mouse CD3 Antibody [17A2], Cat# 100236, 0.5 µg per million cells in 100 µL volume, BioLegend, validated for flow cytometry and tested to detect in mouse by the manufacturer, https://www.biolegend.com/en-gb/products/apc-anti-mouse-cd3-antibody-8055.
15. PE anti-mouse CD4 Antibody [GK1.5], Cat# 100408, 0.25 µg per million cells in 100 µL volume, BioLegend, validated for flow cytometry and tested to detect in mouse by the manufacturer, https://www.biolegend.com/en-gb/products/pe-anti-mouse-cd4-antibody-250.
16. FITC anti-mouse CD8a Antibody [53-6.7], Cat# 100706, 1.0 µg per million cells in 100 µL volume, BioLegend, validated for flow cytometry and tested to detect in mouse by the manufacturer, https://www.biolegend.com/en-gb/products/fitc-anti-mouse-cd8a-antibody-153.
17. APC anti-mouse H-2Kb Antibody [AF6-88.5], Cat# 116506, 1.0 µg per million cells in 100 µL volume, BioLegend, validated for flow cytometry and tested to detect in mouse by the manufacturer, https://www.biolegend.com/nl-be/products/fitc-anti-mouse-h-2kb-antibody-1748?GroupID=BLG2539.
18. Purified anti-mouse IFN-γ Antibody [R4-6A2], Cat# 505702, 2.0 µg mL-1, BioLegend, validated for ELISpot assay and tested to detect in mouse by the manufacturer, https://www.biolegend.com/nl-be/products/purified-anti-mouse-ifn-gamma-antibody-987.
19. Ultra-LEAF™ Purified anti-mouse CD8a Antibody [53-6.7], Cat# 100764, 10.0 mg kg-1, BioLegend, validated for in vivo application and tested to detect in mouse by the manufacturer, https://www.biolegend.com/en-gb/products/ultra-leaf-purified-anti-mouse-cd8a-antibody-7731.
20. In Vivo MAb anti-mouse PD-L1(B7-H1) [Clone: 10F.9G2], Cat# BE0101, 10.0 mg kg-1, BioXcell, validated for in vivo application and tested to detect in mouse by the manufacturer, In Vivo MAb anti-mouse PD-L1(B7-H1) [Clone: 10F.9G2], Cat# BE0101, 10.0 mg kg-1, BioXcell, validated for in vivo application and tested to detect in mouse by the manufacturer. https://bxcell.com/product/m-pdl-1/.

Eukaryotic cell lines

Policy information about cell lines and Sex and Gender in Research

Cell line source(s)	The mouse CT26, RAW264.7, B16-OVA and 4T1 cells were purchased from China Type Culture Collection, supplied by the American Type Culture Collection (ATCC).
Authentication	The parental CT26, RAW264.7, B16-OVA and 4T1 cell lines were authenticated by the American Type Culture Collection (ATCC).
Mycoplasma contamination	All cell lines in this study get tested without mycoplasma contamination.
Commonly misidentified lines (See ICLAC register)	No misidentified lines.

Animals and other research organisms

Policy information about studies involving animals; ARRIVE guidelines recommended for reporting animal research, and Sex and Gender in Research

Laboratory animals	BALB/c mice and C57 mice (5 weeks old) were purchased from medicine center of Yangzhou university (Yangzhou, China). All animals were housed with the light cycle of 12 h: 12 h, ambient temperature at 22 degree Celsius, and relative humiditrange between 40-70%.
Wild animals	The study did not involve wild animals.
Reporting on sex	The mice used in the CT 26 and B16-OVA tumor model in this study were male, and the mice used in the 4T1 breast cancer model were female.
Field-collected samples	The study did not involve any samples collected from field.
Ethics oversight	All mice were obtained from medicine center of Yangzhou university (Yangzhou, China). All animal work was approved by the Institution Animal Care and Use Committee of Nanjing University (IACUC-D2202156) and conducted in accordance with the principles of the Association for Assessment and Accreditation of Laboratory Animal Care International (AAALAC).

Note that full information on the approval of the study protocol must also be provided in the manuscript.

Flow Cytometry

Plots

Confirm that:

☒ The axis labels state the marker and fluorochrome used (e.g. CD4-FITC).

☒ The axis scales are clearly visible. Include numbers along axes only for bottom left plot of group (a 'group' is an analysis of identical markers).

☒ All plots are contour plots with outliers or pseudocolor plots.

☒ A numerical value for number of cells or percentage (with statistics) is provided.

Methodology

Sample preparation	Cell samples were collected from primary and distant murine subcutaneous tumor tissues, digested with collagenase/dispase solution, and passed through single-cell filters.
Instrument	FACS data was collected using BD FACS Calibur flow cytometer.
Software	FACS data was collected using BD FACS Calibur and analyzed using Flow Jo v10.8.1.
Cell population abundance	FACS analysis was performed on each sample to a total cell number between 50,000 to 1,000,00 events with a threshold of 10,000 to increase quality of samples per event. Each gated population was sorted so that at least 500 cells for the furthest gated cell population was recorded to obtain satisfactory percentage of the cell population. FACS quality was also ensured using compensation controls and FMO controls to verify that observed and gated populations were accurate and distinct.
Gating strategy	All of the flow cytometry experiments were adopted with this sample treatment method and gating strategy. After incubated with various antibodies, cells were fixed by 4% paraformaldehyde and then analysed via flow cytometry. During the the running process, Forward Scatter (FSC) and Side Scatter (SSC) dot maps were established, the voltage was adjusted to ensure that all the events were within the visible range of the dot maps. Then, the events with appropriate FSC (200-600) and SSC (200-600) were gated and collected. Those events with low FSC/low SSC and low FSC/high SSC were abandoned, which mainly represented cell debris and air bubbles. Followed by cell type specific gating using fluorescently labeled antibodies.

☒ Tick this box to confirm that a figure exemplifying the gating strategy is provided in the Supplementary Information.
